# Transplantation of Human Tumours into Mouse-brain

**DOI:** 10.1038/bjc.1954.46

**Published:** 1954-09

**Authors:** G. Lumb

## Abstract

**Images:**


### TRANSPLANTATION OF HUMAN TUMOURS INTO MOUSE-BRAIN.

G. LUMB.

*From the Imperial Cancer Research Fund Laboratories at the Royal College of Surgeons, London, and the Department of Pathology, Westminster Medical School, London.*

Received for publication July 22, 1954.

THE maintenance of human tumours under artificial conditions is a problem which has tested experimental workers for many years. Heterologous transplantation into different animals has been considered by most observers as unlikely to succeed for a variety of reasons, among which may be quoted the essential specificity of the protein make-up of cells in any particular animal species and the immune reaction leading to their destruction which is likely to be set up on their introduction into a heterologous strain. Failure of growth in new surroundings is characterised by lymphocytic infiltrations, proliferations of fibroblasts, collagen formation and failure of vascularisation. Loeb (1930) felt that death of a transplant within a few days of inoculation was due to immune responses by connective tissue elements at the site rather than secondary development of humoral antibodies. Sites for inoculation were sought where blood supply and connective tissue capable of immediate granulomatous response against a foreign body were minimal and where it might be hoped that foreign cells would become established sufficiently in their new environment to withstand vascularisation and subsequently to gain nourishment from it in order to proliferate.

As early as 1873 Van Dooremaal suggested that the anterior chamber of the eye might provide a suitable site for inoculation and in 1884 Zahn attempted to transplant a human chondroma in this way. It became infiltrated by lymphocytes and was absorbed.

Subsequent experiments seemed to confirm the early fears of failure and most workers formed the view that whilst homologous transplantation—that is transference into a like species—was sometimes possible, particularly when a rapidly growing tumour was employed, heterologous transplantation would prove uniformly unsuccessful.

In 1937 Smirnova reported successful transference of a human mammary carcinoma, and Greene with co-workers, in a series of papers from 1938 up to the present time, has described successful results of heterologous transplantation of human tumours, usually into the anterior chamber of the eyes of guinea pigs, although other animals such as rabbits have been used.

The results obtained by Greene have been more successful than those reported by other workers and he has developed the thesis that the ability of tumours to survive in heterologous species may be a measure of the ability of the malignant cell to metastasise. He recognises two groups of tumours from the point of view of transplantability, the difference between which is not based on the morphology of the neoplasm, but on the results of their behaviour in the patient. Thus he describes two groups of tumours, one of which contains hetero-transplantable material obtained from patients whose disease follows a rapidly fatal course, and the other consists of material from patients who live for considerable periods of time. He goes further to suggest that when a tumour becomes capable of metastasis it also becomes possible to transplant it successfully into heterologous species. (Greene, 1952).

This view is supported by Lemon and Parsons (1952) who base their opinion on a small series of cases ; but Towbin (1951*a*) reporting on the results of one hundred different human tumours, and thirty-eight different animal tumour transplants, was unable to confirm these findings to his satisfaction and concluded that relatively few human tumours, even though clinically malignant, can be transplanted successfully to the anterior chamber of the guinea pig's eye. He felt that this factor precludes the use of the technique as a practical measure for differentiating benign from malignant tumours. Morris, McDonald and Mann (1950) agree with these conclusions. After attempting to transplant forty different human malignant tumours they decided that successful heterotransplants into the anterior chamber of the eye can " seldom be accomplished." It was their view, however, that more successful results could be obtained if the tumour was introduced into the lens.

Although the anterior chamber of the eye has been the most popular site for attempted heterotransplantation, many other anatomical areas have been used, most of which have proved unsuccessful, with the exception of the brain in mice and the cheek pouch in hamsters. Greene (1951) has reported successes with mouse-brain inoculation and Lemon and Parsons (1952) have reported early successes in the hamster cheek pouch.

In addition to the search for a suitable site for tumour transplantation various attempts have been made to assist growth in the new surroundings. Most of these attempts have been designed to counteract or inhibit the immune reaction on the part of the host. Pre-operative irradiation of the host animal in order to reduce circulating lymphocytes has been tried by Toolan (1951) with apparent improvement in the transplanted tumour growth. Cortisone and A.C.T.H. have been administered but so far few reports of results are available. Several workers, among whom may be mentioned Toolan (1951), Lemon and Parsons (1952), and Dobyns and Lennon (1952), have tried serial transplantation of human tumours, and the last named authors describe a single case of a thyroid carcinoma which they have maintained by serial transplantation over a period of $3\frac{1}{2}$ years. In addition they record the interesting fact of an increasing degree of histological differentiation in the tumour during this period.

It has been suggested that the criteria for assessing continued viability of a tumour in its new surroundings are not sufficiently accurate and that the demonstration of cells which are alive as judged by histological means, or an increase in total size of a tumour, does not necessarily indicate a " take." Towbin (1915*b*) has pointed out in relation to transplantation to guinea pig anterior chamber that gross appearances may be deceptive and must be substantiated by histological studies. He has suggested the analysis of transplantation results in three stages.

1. A stage of suspension which may be compared to the conditions of tissue culture where the aqueous humour acts as a temporary nutritional medium.

2. A stage of nidation where the transplanted tumour elements become integrated with the vascular system of the new host. At this stage death may occur, or if the transplantation is successful, growth will ensue. In many cases Towbin (1951*b*) considers that a period of growth arrest may develop where no change takes place in the tumour transplant and this stage may be maintained for very long periods.

3. A stage of growth when, if transplantation has been completely successful, the tumour proliferates.

The use of mammalian or avian brain as a focus for the transplantation of heteroplastic tissue seems to have been recorded first by Shirai in 1921 who reported briefly that tumours from rats had been grown successfully in the brains of chickens, pigeons and other animals. Murphy in 1926 presented a careful account of the transplantation of mouse sarcoma and carcinoma into rat brain. He recorded satisfactory and rapid growth up to periods of 3 to 4 weeks.

It was his opinion that failure of growth of a heteroplastic graft was not due to any lack of suitable nutrient material or to the formation of specific antibodies. The evidence available was, he felt, that the cellular reaction was of the first importance in determining the fate of a heteroplastic graft. The accumulation of small round cells around foreign material introduced into the higher animals has been well recorded, and it was Murphy's (1926) view that where this reaction is absent as in the embryo or in the brain, the heteroplastic graft meets with no resistance and its rate of growth is at least as great as it would be in the native host. He felt that all his evidence pointed to the importance of the lymphoid type of cell in the resistance to foreign tissue, and he performed experiments of reducing total lymphocytes by X-irradiation and by adding lymphocytic material, usually in the form of fresh spleen, along with the implanted tissue, the results of which he felt confirmed his theory.

In view of the relatively small number of reports in the literature of the results of heterotransplantation of human tumours it was decided to commence a series of experiments. A start has been made using the mouse-brain for inoculation as this site appeared to have been the least studied, but later the anterior chamber in guinea pigs has been used and more recently still, experiments have been commenced in which the cheek-pouch of the hamster has been utilised. This presentation is a report of the results obtained following transplantation of sixty different human tumours into mouse-brain and the account will be confined largely to changes taking place during the first 4 to 5 weeks following inoculation.

### TECHNIQUE.

Malignant human tumours are obtained from Westminster Hospital. The portion for inoculation is selected under aseptic conditions in or near the operating theatre immediately after removal. An attempt is made to take a piece of tumour with as little stroma as possible and also to avoid obvious areas of necrosis or haemorrhage. The specimen is placed in a sterile container and transported at once to the laboratories of the Imperial Cancer Research Fund at the Royal College of Surgeons of England. The time interval at best is in the region of 20 minutes, but delays up to an hour are sometimes unavoidable. Speed of transference is of vital importance in this operation and for this reason a series of experiments are to be performed where inoculation is done on the spot and the animals subsequently transferred to the laboratory. So far no attempt has been made to maintain the tumour at any particular temperature during the interval between its removal and its inoculation.

The animals used are mice of up to 10 weeks of age. In the light of the experience of previous workers, young animals are preferred, but in view of the irregular availability of human tumour material it has been found impossible to insist on any very particular age. In the early stages of the experiment pure bred C3H mice were used, but later mixed strains have been accepted with no apparent effect on results.

Basal anaesthesia is employed (0·1 c.c. of a 0·016 per cent solution of nembutal per 10 g. weight of mouse) and is administered intraperitoneally. This gives quite rapid deep anaesthesia which lasts for 2 hours. It has the distinct advantage that a batch of mice can be prepared when the human operation is about to commence so that the experimental animals are ready for inoculation as soon as the tumour is available, thus cutting down the transference time interval.

The method of inoculation is to shave the skin of the mouse's head over the vertex and to make a small incision down to the bone. In the earlier experiments a small triangular bone flap was then raised which could be replaced at the end of the operation. This has been found unnecessarily complicated and time consuming, so that now a small hole is made with the end of a corneal scalpel. The skull of the mouse is thin yet rigid so that little difficulty is experienced in applying the correct pressure to produce a hole without damaging the underlying brain. Bleeding is usually minimal and can be stopped quickly by gentle pressure with a gauze swab.

A small fragment of tumour is then introduced into the brain either on the end of a blunted hypodermic needle, or, after rough mincing with scissors, it can be drawn into the needle and either pushed in with the needle stylette or forced in by pressure from an ordinary syringe. A No. 18 gauge hypodermic needle preferably provided with a guard at 3 mm. from its blunted point, has been found the most useful instrument for this procedure. Two points of importance emerge in connection with the technique of tumour introduction. Firstly, only a very tiny fragment of tumour material should be used. Larger pieces tend to undergo rapid necrosis. It is clearly impossible to define an exact size but the inoculum either minced or unminced should represent a piece of tumour certainly not more than 1 mm. in diameter. The second point of importance is to make sure that when the needle is removed from inside the skull, the tumour inoculum does not also come out. No great difficulty is experienced in achieving this, but a small practical point of importance is to make the line of needle entry oblique to the skull hole.

When inoculation is complete the skin is drawn together over the wound and sealed with collodion. The mouse is then replaced in its cage and within 12–18 hours returns to apparent complete normality. Mortality among the mice as a result of the operation is related directly to operation technique. In the earlier part of the experiment 20 mice were inoculated in each batch and 2 or 3 fatalities within the first 3 days were common. Now 10 mice are inoculated in each batch and a fatality is a matter of extreme rarity.

A portion of the tumour inoculated is fixed and examined histologically. When experiments were commenced an attempt was made to select a suitably cellular area of tumour by performing immediate frozen sections. No apparent advantage resulted from this, and it had the disadvantage of causing delay in inoculation so that it has now been abandoned.

The whole procedure of inoculation is performed using aseptic precautions. The operation takes place in a sterile chamber and sterile rubber gloves are worn by the operator. Until fully recovered from the anaesthetic mice are segregated, but after 24 hours, inoculated batches are kept together under normal animal house conditions.

### RESULTS.

Throughout the experiment close observation of the inoculated mice has been maintained in order to observe any signs of cerebral compression resulting from tumour growth in the skull. No such signs appeared and it was early decided to kill the mice after specific intervals in order to examine the brains. The times selected for observation were at first 10, 15 and 21 days, but later when it was realised that in the absence of complete failure something would be seen in most cases at 10 days, the times were changed to 15, 21 and 28 days. More recently still, mice are killed at 15, 28 and 35 days. This report will be confined to observations made at these times, but from each batch some mice are sacrificed for serial transplantation experiments whilst others are kept indefinitely.

In the early stages of the experiment when technique had not been fully developed each tumour specimen was inoculated into 20 mice, but in order to economise in storage space 10 mice have been used since the time when operative mortality became negligible. Two mice are killed on each selected day and the remainder of the batch used for other purposes or kept indefinitely. The commonest cause of an operative death is haemorrhage into the brain substance. When the animal is killed the head is removed and a post-mortem performed on the remainder of the body. No example of metastasis from inoculated tumours has been discovered.

The skin is carefully stripped from the skull and although even by 10 days considerable or complete healing at the site of inoculation is found, it is usually quite easy to see where the tumour has been put into the skull up to several weeks afterwards. Next the bone is removed and the brain carefully extracted intact. Again it is usual to be able to see macroscopically the point of inoculation on the surface of the cerebrum. The cerebellum and pons are removed and in some cases where the tumour is obvious the cerebellum is trimmed towards the site. The remaining specimen is then fixed routinely for 24 hours in Formol-acetic (Suza's fixative as modified by Heidenhein). This fixative is found suitable as it preserves good cytological detail and at the same time tends to make the brain firm and so facilitates subsequent section cutting. Serial sections are then cut from each specimen although it is usual for the section cutter to be able to recognise the point in the block where he begins to cut through the tumour area. Every fifth section has been retained and of these, alternate ones are stained routinely by haematoxylin and eosin, leaving the intervening sections for further investigation if required.

The tumours which have been used up to date are listed in Table 1 and are seen to be made up of sixty different human cases which fall into the following groups:

| | Cases. |
|---|---|
| Carcinoma of breast . . . . . | 27 |
| Squamous cell carcinomas . . | 8 |
| Melanomas . . . . . . | 6 |
| Ovarian carcinomas . . . . . | 6 |
| Carcinomas of gastro-intestinal tract . | 8 |
| Sarcomas . . . . . . . | 3 |
| Carcinoma of lung . . . . | 1 |
| Carcinoma of kidney . . . . | 1 |

TABLE I.

| No. | Age. | Sex. | Tumour type. | Metastases present or absent (+ or −). | Tissue used for inoculation. | No. of days up to which definite cells seen in mouse-brain. |
|---|---|---|---|---|---|---|
| 1 | 55 | F | Carcinoma breast | − | Primary tumour | Failure |
| 2 | 43 | F | ,, ,, | + but pre-op irr. | ,, ,, | ,, |
| 3 | 66 | F | ,, ,, | − | ,, ,, | ,, |
| 4 | 67 | F | ,, ,, | + Glands in axilla | ,, ,, | ,, |
| 5 | 62 | F | ,, ,, | + ,, ,, ,, | ,, ,, | 364 days |
| 6 | 45 | F | ,, ,, | + ,, ,, ,, | ,, ,, | 28 ,, |
| 7 | 48 | F | ,, ,, | − | ,, ,, | 15 ,, |
| 8 | 43 | F | ,, ,, | − | ,, ,, | Failure |
| 9 | 69 | F | ,, ,, | + Glands in axilla | ,, ,, | 21 days |
| 10 | 45 | F | ,, ,, | + ,, ,, ,, | ,, ,, | 15 ,, |
| 11 | 59 | F | ,, ,, | − | ,, ,, | Failure |
| 12 | 65 | F | ,, ,, | + ,, ,, ,, | ,, ,, | 15 days |
| 13 | 62 | F | ,, ,, | − | ,, ,, | Failure |
| 14 | 54 | F | ,, ,, | − | ,, ,, | 21 days |
| 15 | 62 | F | ,, ,, | − | ,, ,, | Failure |
| 16 | 47 | F | ,, ,, | + Glands in axilla | ,, ,, | 21 days |
| 17 | 55 | F | ,, ,, | + ,, ,, ,, | ,, ,, | 21 ,, |
| 18 | 57 | F | ,, ,, | + ,, ,, ,, | ,, ,, | 28 ,, |
| 19 | 49 | F | ,, ,, | + ,, ,, ,, | ,, ,, | 28 ,, |
| 20 | 72 | F | ,, ,, | + ,, ,, ,, | ,, ,, | Failure |
| 21 | 47 | F | ,, ,, | − | ,, ,, | 21 days |
| 22 | 43 | F | ,, ,, | + ,, ,, ,, | ,, ,, | 35 ,, |
| 23 | 52 | F | ,, ,, | + ,, ,, ,, | ,, ,, | 28 ,, |
| 24 | 45 | F | ,, ,, | + ,, ,, ,, | ,, ,, | 28 ,, |
| 25 | 45 | F | ,, ,, | + ,, ,, ,, | Metastasis | Failure |
| 26 | 57 | F | ,, ,, | − | Primary tumour | 28 days |
| 27 | 61 | F | ,, ,, | + ,, ,, ,, | Metastasis | Failure |
| 28 | 49 | M | Squamous cell carcinoma | Rec. ca. penis | Primary tumour | 28 days |
| 29 | 56 | M | Ditto | + Glands from tongue | Metastasis | 21 ,, |
| 30 | 48 | M | ,, | + Glands from larynx | ,, | 21 ,, |
| 31 | 66 | M | ,, | + Glands from tongue | ,, | 21 ,, |
| 32 | 51 | F | ,, | + Glands cervix uteri | ,, | 21 ,, |
| 33 | 42 | M | ,, | − Skin carcinoma | Primary tumour | 15 ,, |
| 34 | 64 | M | ,, | + Glands from tongue | Metastasis | 21 ,, |
| 35 | 52 | M | ,, | + ,, ,, ,, | ,, | 21 ,, |
| 36 | 31 | F | Melanoma | − | Primary tumour | Failure |
| 37 | 61 | M | ,, | − | ,, ,, | ,, |
| 38 | 52 | F | ,, | + Glands from groin | Metastasis | 21 days |
| 39 | 63 | F | ,, | + ,, ,, ,, | ,, | 28 ,, |
| 40 | 57 | F | ,, | + ,, in ,, | ,, | 28 ,, |
| 41 | 58 | M | ,, | + ,, ,, ,, | ,, | 28 ,, |
| 42 | 31 | F | Carcinoma ovary | + Omental spread | Primary tumour | Failure |
| 43 | 37 | F | ,, ,, | + Generalised | ,, ,, | 15 days |
| 44 | 61 | F | ,, ,, | + Krukenberg | Metastasis | 15 , |
| 45 | 50 | F | ,, ,, | + Generalised | ,, | 28 ,, |
| 46 | 49 | F | ,, ,, | + Omental spread | ,, | 15 ,, |
| 47 | 40 | F | ,, ,, | + Liver | ,, | 21 ,, |
| 48 | 64 | M | ,, stomach | + Adjacent glands | ,, | 21 ,, |
| 49 | 73 | F | ,, ,, | + Glands | Primary tumour | Failure |
| 50 | 73 | F | Carcinoma rectum | + ,, | ,, ,, | ,, (inf.) |
| 51 | 63 | F | ,, ,, | + ,, | ,, ,, | ,, ,, |
| 52 | 61 | M | ,, ,, | + ,, | Metastasis | 35 days |
| 53 | 62 | M | Carcinoma colon | + Adjacent glands | ,, | 21 ,, |
| 54 | 62 | M | ,, ,, | + ,, ,, | Primary tumour | 21 ,, |
| 55 | 49 | F | ,, ,, | + ,, ,, | ,, ,, | 28 ,, |
| 56 | 22 | M | Osteogenic sarcoma | − | ,, ,, | Failure |
| 57 | 79 | M | Fibrosarcoma | − | ,, ,, | 28 days |
| 58 | 23 | M | Osteogenic sarcoma | − | ,, ,, | 15 ,, |
| 59 | 54 | M | Carcinoma lung | − | ,, ,, | Failure (inf.) |
| 60 | 56 | M | Carcinoma kidney | − | ,, ,, | 35 days |

They are arranged in the table to show the age, sex and diagnosis of the patient from whom the tumour was taken, in addition to the result achieved on transplantation expressed in terms of number of days after inoculation at which tumour cells could be recognised by histological means.

In those cases where tumour cells are found they reproduce closely the histological appearances they presented when growing in human tissues and are readily recognisable (Fig. 6–15). The site of inoculation in the brain is clear cut and shows a sharp line of demarcation between inoculum and normal brain. The resulting growths following implantation tend to produce a discrete rounded nodule (Fig. 1) pushing aside the surrounding brain rather than infiltrating it. At 10 and 15 day periods necrosis of some of the tumour cells is seen. Sometimes maximum cell death is central in a nodule (Fig. 2) and more rarely it is the peripheral cells of a tumour clump which are mainly affected (Fig. 3). This death of cells is frequently associated with an infiltration of polymorphonuclear leucocytes (Fig. 3).

In brains examined at 21 days where some tumour cells have survived, inflammatory cellular infiltration is minimal both in the tumour nodule itself and also about its edges. Small vessels in the vicinity however, tend to be dilated and show perivascular " cuffing " by small round cells of lymphocyte type (Fig. 4). Frequently small vessels are seen plugged with lymphocytes. At 28 and 35 day periods in most of the cases examined in this series histologically identifiable

### EXPLANATION OF PLATES.

FIG. 1.—Discrete rounded nodule of tumour well demarcated from surrounding brain. Transplant from metastasis from carcinoma ovary after 15 days. H. & E. × 55.
FIG. 2.—Central necrosis in group of cells from a fibrosarcoma after 15 days. H. & E. × 110.
FIG. 3.—Peripheral death of cells with well marked polymorphonuclear leucocyte proliferation from a squamous cell carcinoma 10 days after transplantation. H. & E. × 100.
FIG. 4.—Lymphocytic infiltration around small vessels at edge of inoculation area. H. & E. × 250.
FIG. 5.—Zone of inoculation where malignant cells have disappeared. Vascularisation and early fibrosis have taken place. H. & E. × 120.
FIG. 6*a*.—Carcinoma of breast, Stage II before inoculation. H. & E. × 260.
FIG. 6*b*.—Inoculated tumour in Fig. 6*a* after 21 days. H. & E. × 260.
FIG. 7*a*.—Carcinoma of breast, Stage II before inoculation. H. & E. × 250.
FIG. 7*b*.—Inoculated tumour in Fig. 7*a* after 15 days. H. & E. × 350.
FIG. 8*a*.—Carcinoma of breast, Stage II before inoculation. H. & E. × 240.
FIG. 8*b*.—Inoculated tumour in Fig. 8*a* after 21 days. H. & E. × 280.
FIG. 9*a*.—Metastasis in gland from squamous cell carcinoma before inoculation. H. & E. × 260.
FIG. 9*b*.—Inoculated tumour in Fig. 9*a* after 21 days. H. & E. × 80.
FIG. 9*c*.—Inoculated tumour in Fig. 9*a* after 21 days showing mitotic figure. H. & E. × 650.
FIG. 10*a*.—Metastasis in gland from melanoma before inoculation. H. & E. × 280.
FIG. 10*b*.—Inoculated tumour in Fig. 10*a* after 29 days. H. & E. × 280.
FIG. 11*a*.—Metastasis in gland from melanoma before inoculation. H. & E. × 280.
FIG. 11*b*.—Inoculated tumour in Fig. 11*a* after 21 days. H. & E. × 280.
FIG. 11*c*.—Inoculated tumour in Fig. 11*a* after 15 days invading ventricle. H. & E. × 280.
FIG. 12*a*.—Metastasis in ovary (Krukenberg) before inoculation. H. & E. × 280.
FIG. 12*b*.—Inoculated tumour in Fig. 12*a* after 15 days. Note mitotic figures. H. & E. × 280.
FIG. 13*a*.—Peritoneal metastasis from carcinoma of ovary before inoculation. H. & E. × 320.
FIG. 13*b*.—Inoculated tumour from Fig. 13*a* after 10 days. H. & E. × 320.
FIG. 14*a*.—Peritoneal metastasis from carcinoma of stomach before inoculation. H. & E. × 280.
FIG. 14*b*.—Inoculated tumour from Fig. 14*a* after 21 days. H. & E. × 280.
FIG. 15*a*.—Fibrosarcoma before inoculation. H. & E. × 280.
FIG. 15*b*.—Inoculated tumour in Fig. 15*a* after 10 days. H. & E. × 120.
FIG. 16.—Squamous cell carcinoma proliferating over surface of ventricle 21 days after inoculation H. & E. × 350.

1

2

5

Lumb.

6A

6B

8A

8B

10A

10B

12A

12B

Lumb.

14A 14B

15A 15B

Lumb.

3   4

7a   7b

Lumb.

9a

11a

9b

11b

9c

11c

Lumb.

13*a*

13*b*

16

Lumb.

tumour cells have disappeared but when they are present they appear as clumps of cells unassociated with any cellular infiltration.

At the 21 and 28 day period vascularisation of the inoculation zone is the most striking feature. Small capillary blood vessels are seen in abundance and in the areas where tumour cells have disappeared fibroblast proliferation occurs (Fig. 5). Lymphocytic infiltration is not a marked feature of the cerebral inoculations and is restricted to the periphery of the tumour zone. This is in marked contradistinction to the appearances in the anterior chamber of guinea-pig or rabbit eye. In those cases where all tumour cells have been removed, a small focus of fibrous tissue is left from which all vessels gradually disappear leaving a small zone of organised collagen.

The clearly demarcated inoculation site in the brain is always very much larger than the tiny fragment of tumour tissue which is introduced. This zone is not always completely occupied by tumour cells and on many occasions the apparently large size of the site is the result of swelling by tissue fluid.

Certain special features have emerged in relation to specific tumours when introduced into the brain. In the case of breast carcinomas the tissue remaining in the transplantation area when the brains are examined tends to show clumps of well differentiated duct and acinar elements (Fig. 6*b*, 7*b* and 8*b*). At first sight some of these give the appearance of normal duct tissue, but so far no example of normal tissue surviving in the brain has been demonstrable. The explanation would appear to be either that the best differentiated areas survive or that in the new surroundings the cells tend to arrange themselves in patterns which are apparently well differentiated.

Breast tumours are probably the commonest malignant tumour available for transplantation purposes in that they usually occur free from bacterial contamination. They are unfortunately however, not the best suited tumours for this purpose as they tend to occur in their commonest form in close association with fibrous tissue. Twenty-seven have been used in this series and in 16 cases clumps of tumour cells have been found with the appearances described above. Squamous cell carcinomas have reproduced their normal histological appearances very closely in the mouse brain, producing easily recognisable " cell nests " (Fig. 9*b*) showing mitotic figures around the periphery of the cell clumps (Fig. 9*c*).

Malignant melanomas have also produced encouraging results where solid clumps of apparently viable cells with occasional mitoses have been found (Fig. 10*b* and 11*b*) and in one case evidence of what appears to be infiltration into the ventricle is seen (Fig. 11*c*).

Difficulty has been experienced so far with tumours of the gastro-intestinal tract because of bacterial contamination. Two cases of carcinoma of the rectum produced abscesses in the brain on inoculation. In 2 cases of carcinoma of the colon and a case of carcinoma of the stomach metastases were used with some success (Fig. 14*a*, and 14*b*).

Murphy (1926) in reporting his results of transplantation into mouse-brain stressed the fact that when tumours came in contact with the ventricle, rapid death resulted and he recommended that care should be taken to avoid inoculations near these structures. This has not been the experience in these experiments, for in a number of cases foci of tumour cells have been found in close association with the ventricles and in the case mentioned above, a melanoma appears to be directly infiltrating into a small narrowing of one (Fig. 11*c*). One squamous cell carcinoma spread over the surface of the ventricle (Fig. 16) giving the impression that in this particular tumour the ventricular surface provided a particularly suitable medium for tumour cell proliferation.

The results which have been described so far are in relation to normal mice, but twenty of the human tumours were inoculated both into untreated mice and into mice previously treated by a single whole body dose of 500 r delivered by a 220 Kv. generator. This dose was found by a series of experiments to lower the peripheral leucocyte count from an average of 8,000–9,000 per cu. mm. to 400–500 per cu. mm. With this dose it was decided that a maximal fall of circulating leucocytes could be achieved compatible with absence of untoward effects in the mice. The purpose of treating the mice with pre-operative X-irradiation was to investigate whether or not a diminution of circulating leucocytes would affect transplantation results. Many authors, among whom Murphy (1926) in particular may be quoted, have suggested the importance of the role of the lymphocyte in heterologous transplantation.

The results obtained in this experiment showed no striking variations from those which were achieved in non-irradiated mice. It was decided that any difference did not merit a continuation of the experiment as it had been designed, for the following reason.

The maximum fall of circulating leucocytes occurs 48–60 hours after treatment and remains at the low level for about 7–10 days, when, if the mouse is unaffected, the level rises to normal again. It has been stated already that unlike the anterior chamber of the eye the lymphocytic response in the brain is minimal in the early stages after transplantation, and therefore it would seem likely that at the 3–4 week period when a depressed number of lymphocytes might be an advantage, very little effect would be gained from irradiation. It is felt that irradiation after the tumour has been inoculated would clearly be disadvantageous as it would tend to affect the transplanted tumour cells. For these reasons, therefore, irradiation has been discontinued except in those cases where serial transplantation is being studied.

### DISCUSSION.

In reporting results so far, care has been taken simply to record presence or absence of histologically recognisable tumour tissue without making any attempt to interpret results in terms of " takes." Many authors, including Snell (1954) and Towbin (1951*a*, 1951*b*), have stressed the confusion which arises by recording results simply in terms of " take " or " failure " without making clear the statement. It is obvious that if a transplanted tumour grew as a solid mass to destroy a considerable area of host tissue then no doubt would remain that frank growth was taking place in the transplanted area. The most striking evidence of success of transplantation would be metastasis within the new host, an occurrence which seems unlikely to happen and which has not been claimed even by workers whose results have been most successful.

So far in these experiments no example of metastasis or massive growth has been seen. Only occasionally are mitoses demonstrable and only in 2 cases has there been evidence of infiltration by tumour cells at the edge of the inoculation area. The appearances in " successful " cases have been of isolated areas at the transplantation sites sharply demarcated from adjacent brain showing clumps of cells which by the normal methods of histology appear to be viable. If it were claimed that these cells represent simply a process of suspended animation somewhere between living and dying, but incapable of true proliferation in the manner of a tumour, then very little argument could be brought to the contrary. In the assessment of results so far, however, when careful consideration has been taken of the amount of apparently viable tumour material remaining demonstrable in the period up to 5 weeks after inoculation, the opinion has been formed that the most anaplastic tumours and in particular frank metastases from tumours produce the most active results (Table II).

TABLE II.

| Type of tumour. | No. cases inoculated. | No. cases with cells demonstrable in mouse-brain. | No. of failures. | Notes. |
|---|---|---|---|---|
| Carcinoma of breast | 27 | 16 | 11 | 8 failures were tumours which had not metastasised. 12 successes had already metastasised. |
| Squamous cell carcinoma | 8 | 8 | 1 | Six of the eight cases were metastases. |
| Melanoma | 6 | 4 | 2 | The two failures were primary. The four successes were metastases. |
| Carcinoma of ovary | 6 | 5 | 1 | Four of the five successes were metastases. The failure was a primary. |
| Carcinoma of stomach | 2 | 1 | 1 | |
| Carcinoma of rectum | 3 | 1 | 2 | Both failures were infected primaries. Success was a metastasis. |
| Carcinoma of colon | 3 | 3 | — | All cases had metastasised. |
| Carcinoma of lung | 1 | — | 1 | |
| Carcinoma of kidney | 1 | 1 | — | This was a very anaplastic primary tumour. |
| Sarcomas | 3 | 2 | 1 | The two successes were primaries. |

It has already been stated that Greene has claimed that heterologous transplantation results can be used as an assessment of the virulence of the human tumour in terms of the patient's progress and prognosis. No such immediate claim can be made as a result of these early experiments, but on the other hand it is felt that sufficient promise exists to make worth while a more extensive series of inoculations when the following facts must be established. In the first place it must be made certain whether all normal and benign material fails to survive a transplantation as our early findings and those of other workers suggest. Next, further malignant tumours must be studied in order to establish whether the apparent truth that the degree of malignancy as expressed by behaviour on transplantation can be substantiated.

If the apparent constancy of these results over a relatively small group of cases can be confirmed in large series, then the appearances seen in the transplantation area could conceivably be used in the assessment of a biological test of degree of malignancy, irrespective of whether they represent the true power of growth proliferation or only of suspended animation or nidation.

SUMMARY.

1. A report is given of the results obtained following heterologous transplantation of sixty different human tumours into mouse-brain.

2. The histological appearances during the first 28 to 35 days after inoculation are described.

3. The merits of pre-operative irradiation of the mice are discussed.

4. The tentative suggestion is made that better results are obtained with more rapidly growing and metastasing tumours.

5. Necessity for rapidity of transfer from host to experimental animal is stressed.

This work has been made possible by a grant from the Imperial Cancer Research Fund. My grateful thanks are due to Professor Geoffrey Hadfield for much helpful advice and criticism, to Mr. J. M. Tancock for most able and painstaking assistance with the technical work, and to Mr. E. V. Willmott for the photomicrographs.

### REFERENCES.

Dobyns, B. M., and Lennon, B.—(1952) *Cancer*, **5**, 45.

Greene, H. S. N.—(1938) *Science*, **88**, 357.—(1941) *J. exp. Med.*, **73**, 461, 475.—(1942) *Cancer Res.*, **2**, 649.—(1943) *Ibid.*, **3**, 809.—(1950) *Yale J. Biol. Med.*, **22**, 611.—(1951) *Cancer Res.*, **11**, 529.—(1952) *Cancer*, **5**, 24.

*Idem* and Lund, P. K.—(1944) *Cancer Res.*, **4**, 352.

*Idem* and Murphy, E. D.—(1945) *Ibid.*, **5**, 269.

Lemon, H. M., and Parsons, L.—(1952) *Boston med. Quart.*, **3**, 39.

Loeb, L.—(1930) *Phys. Rev.*, **10**, 547.

Morris, D. S., McDonald, J. R., and Mann, F. C.—(1950) *Cancer Res.*, **10**, 36.

Murphy, J. B.—(1926) *Monogr. Rockefeller Inst. med. Res.*, **21**, 1.

Shirai, Y.—(1921) *Japan med. World*, **1**, 14.

Smirnova, E.—(1937) *Bull. Biol. Méd. exp. URSS.*, **4**, 6.

Snell, G. D.—(1954) 'The Physiopathology of Cancer.' London (Homburger and Fishman), p. 338.

Toolan, H. W.—(1951) *Proc. Soc. exp. Biol., N.Y.*, **77**, 572.

Towbin, A.—(1951*a*) *Cancer Res.*, **11**, 76.—(1951*b*) *Arch. Path.*, **52**, 199.

Van Dooremaal, J. C.—(1873) *V. Graefes. Arch. Ophthal.*, **19**, 359.

Zahn, F. W.—(1884) *Virchows. Arch.*, **95**, 369.


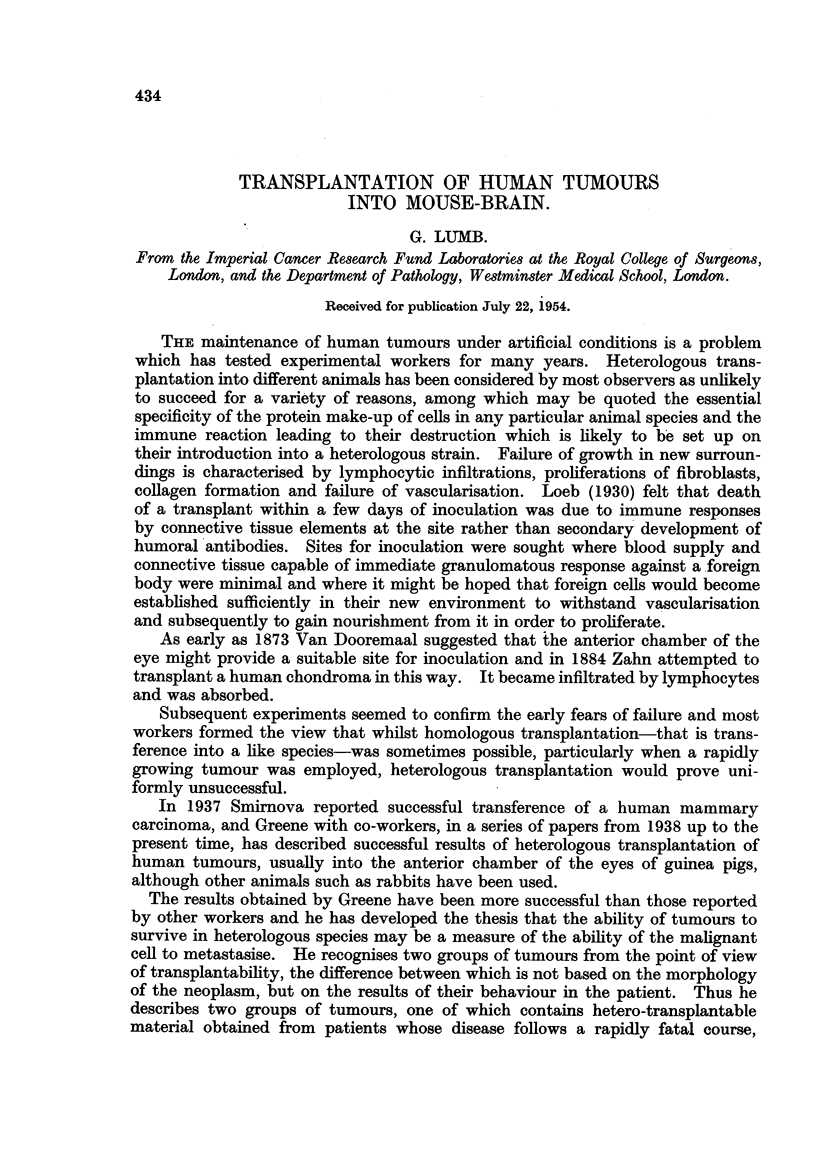

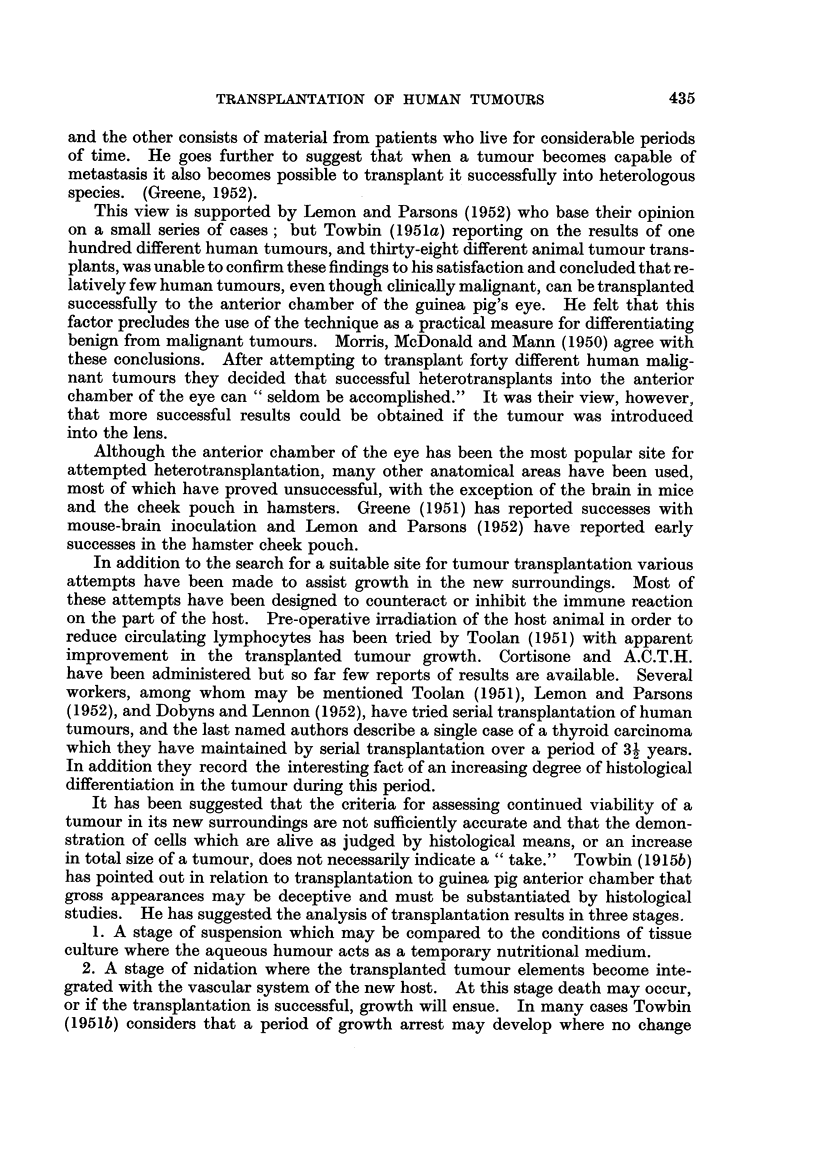

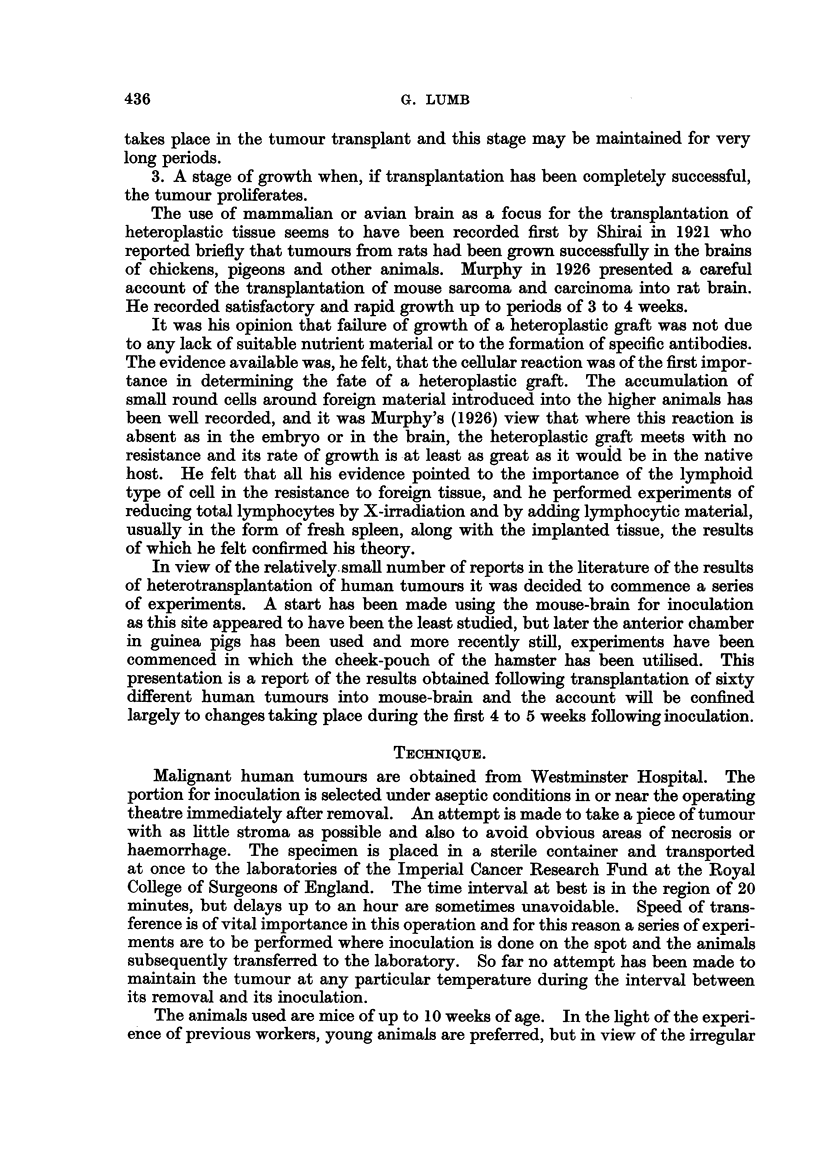

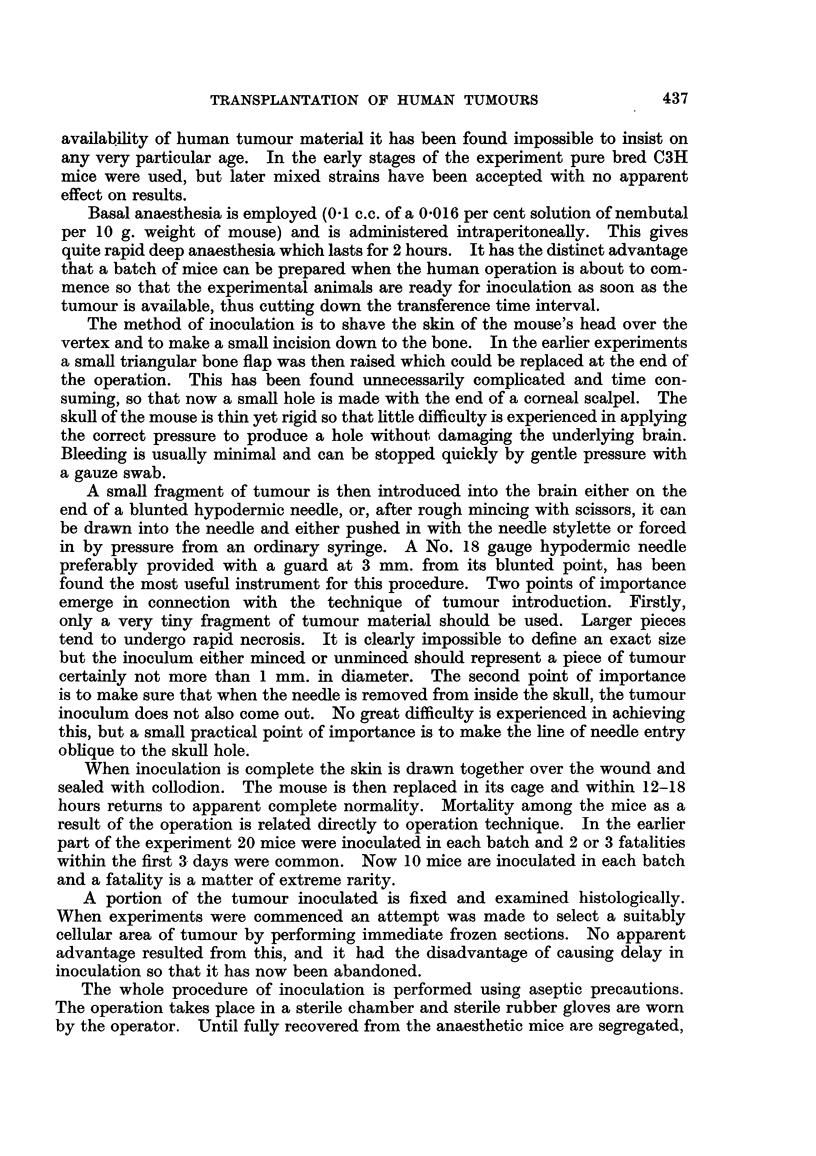

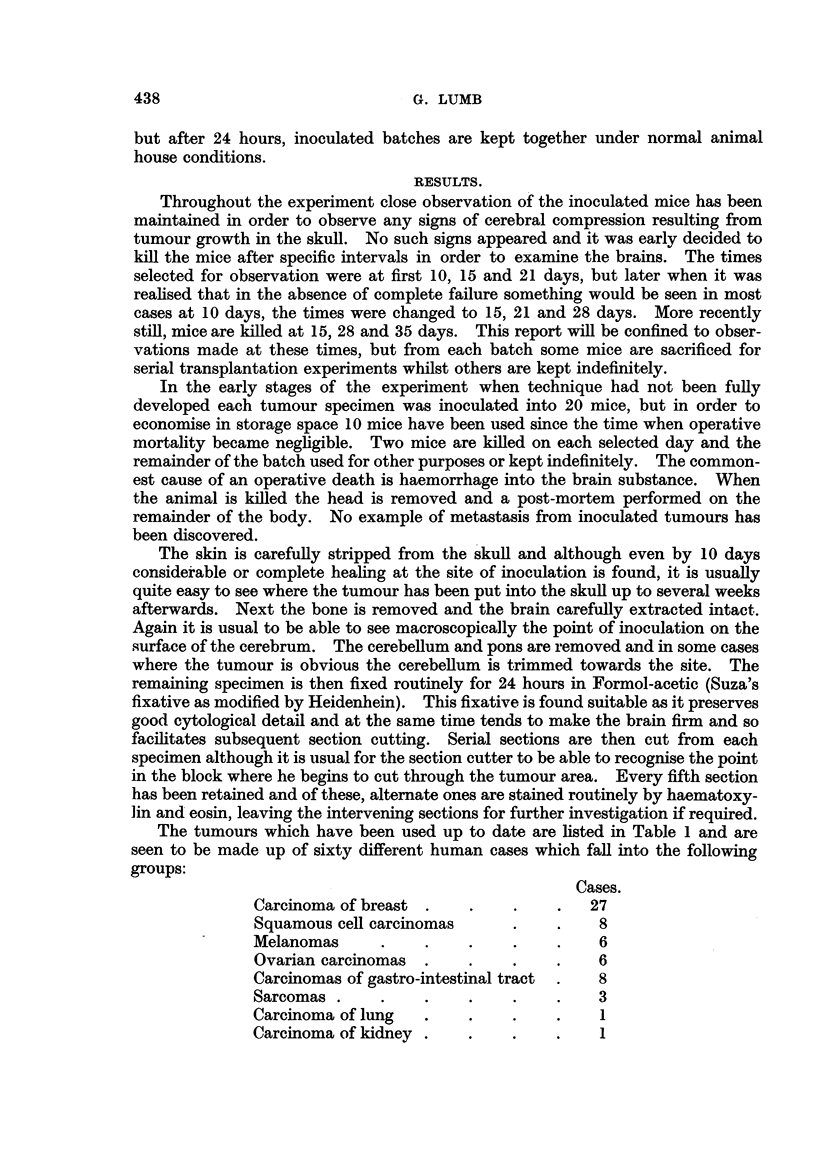

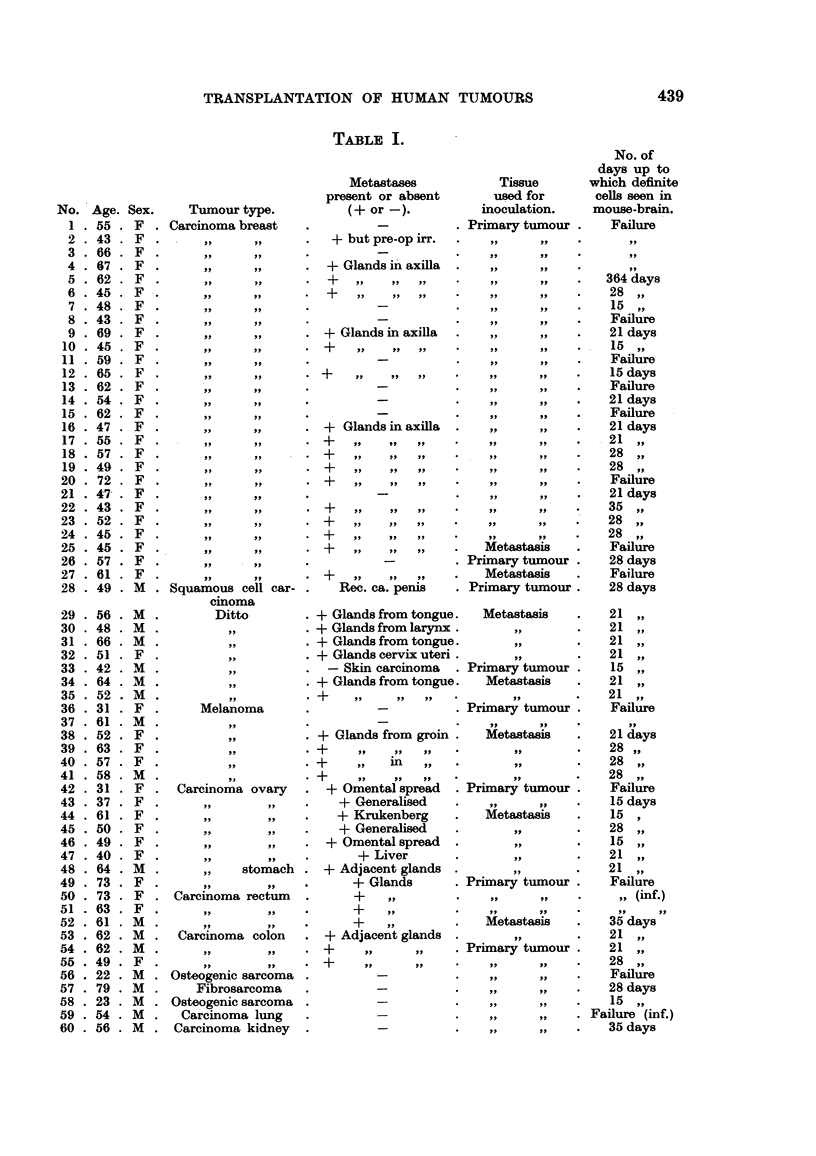

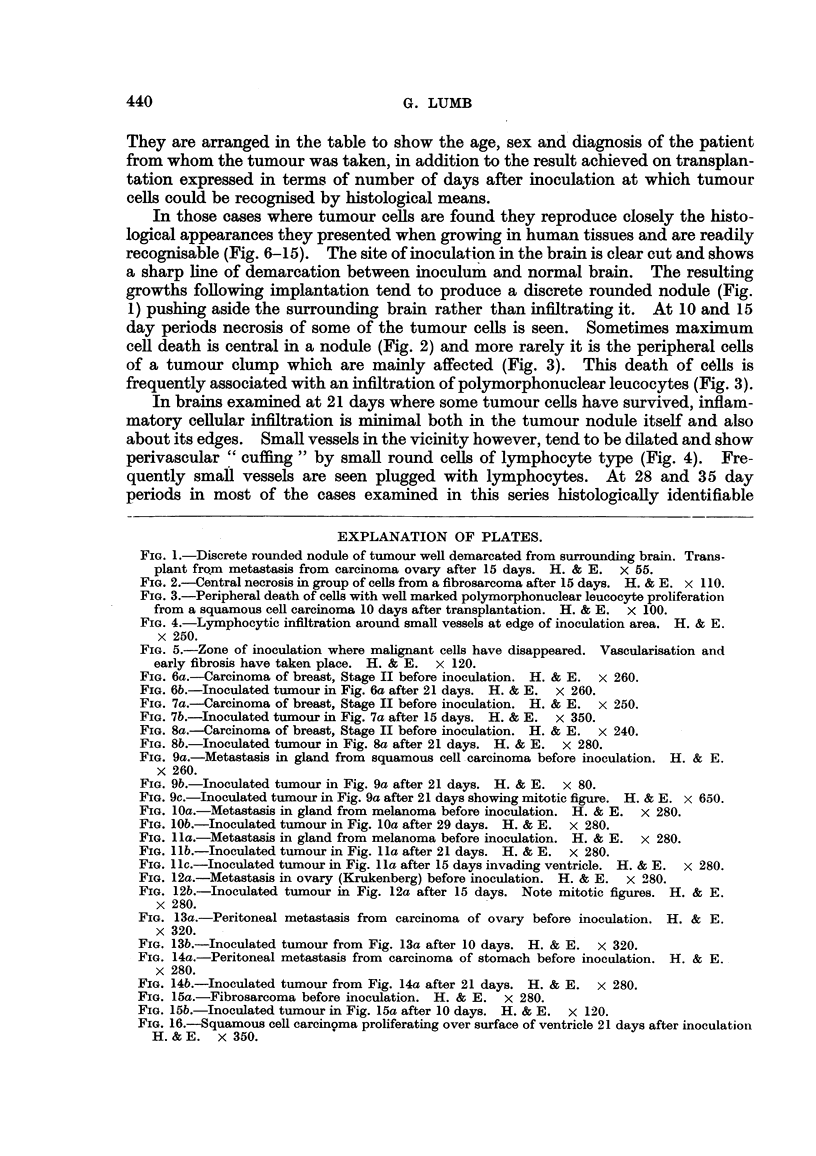

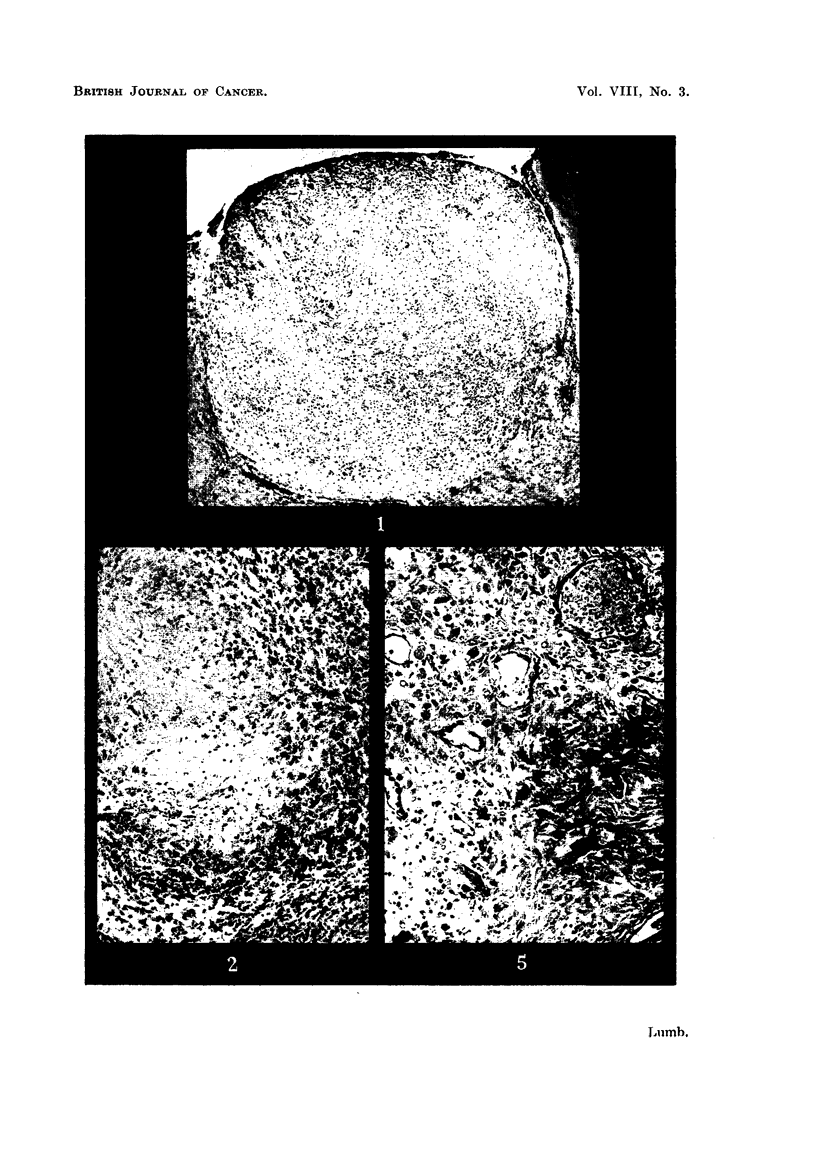

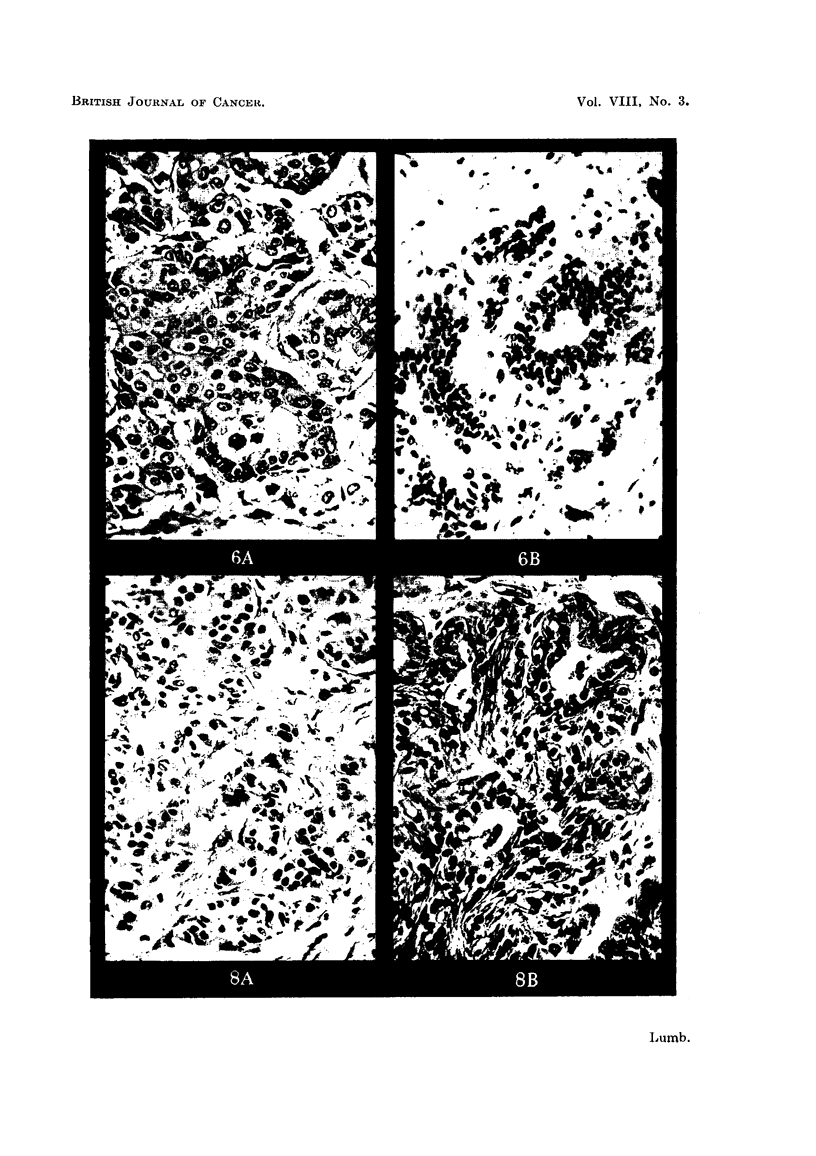

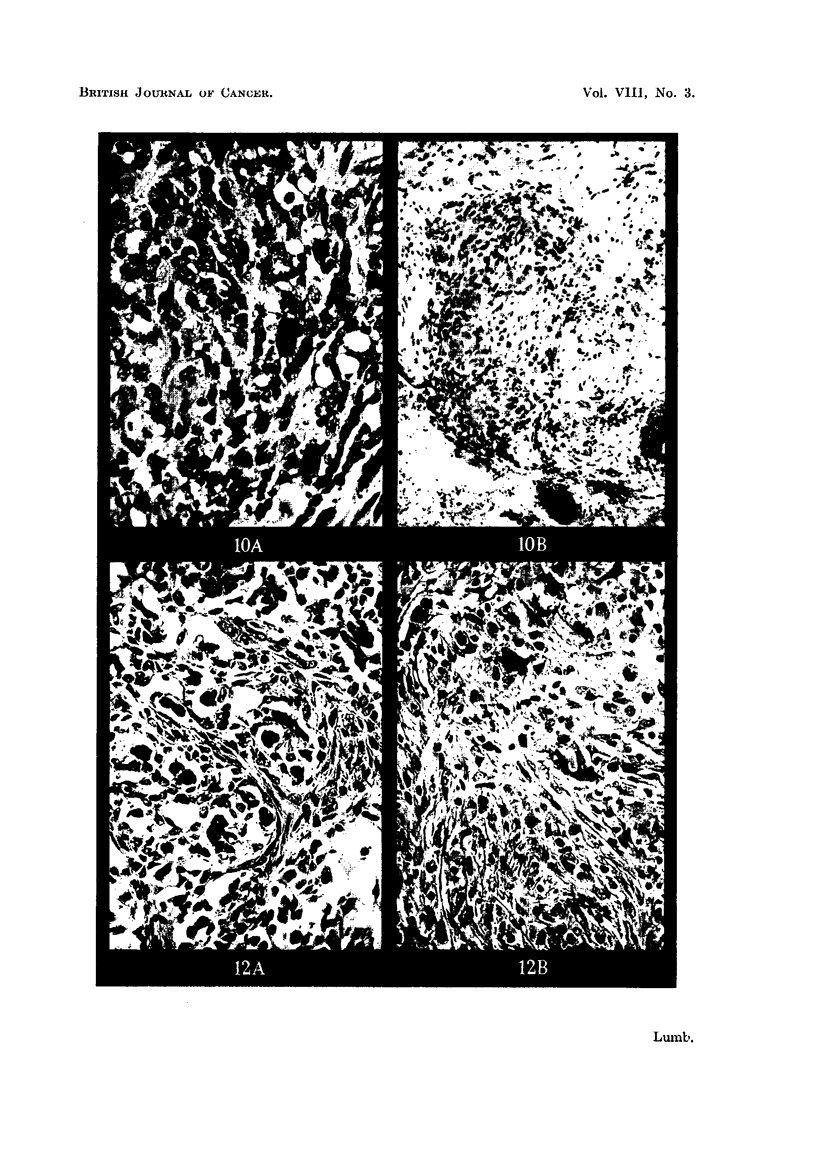

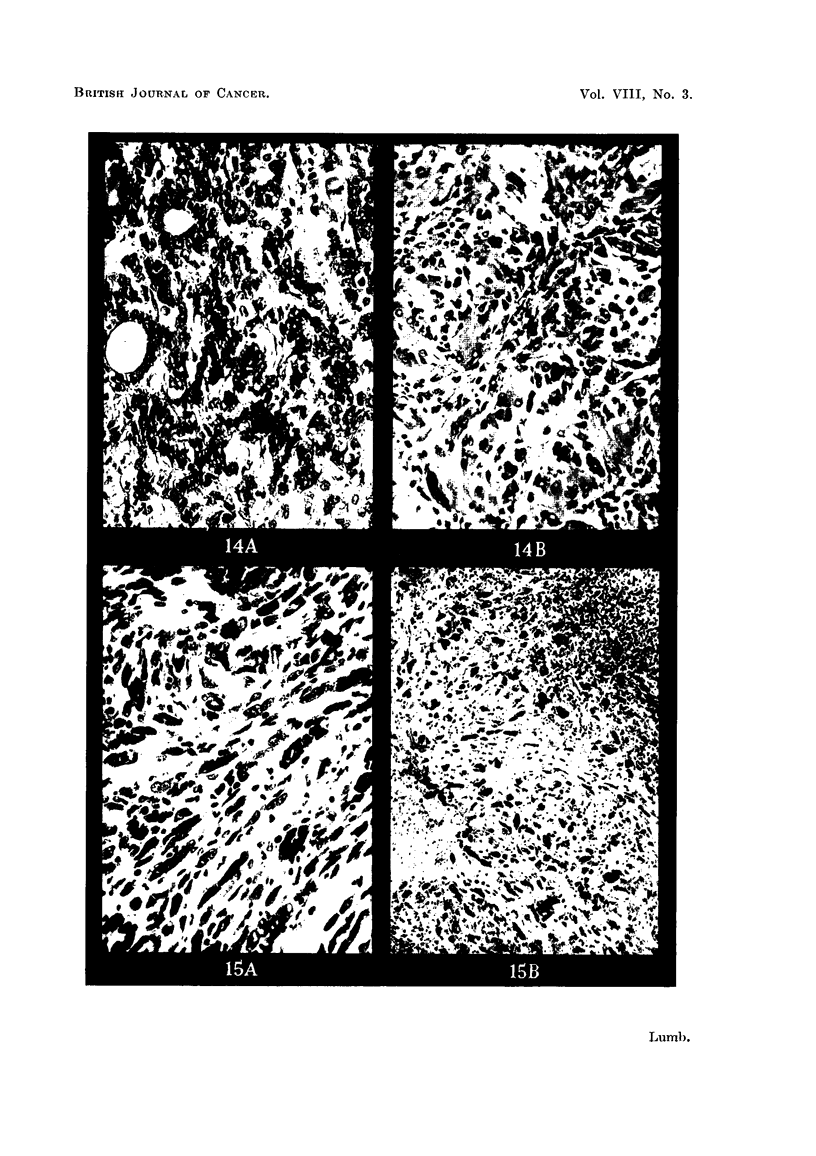

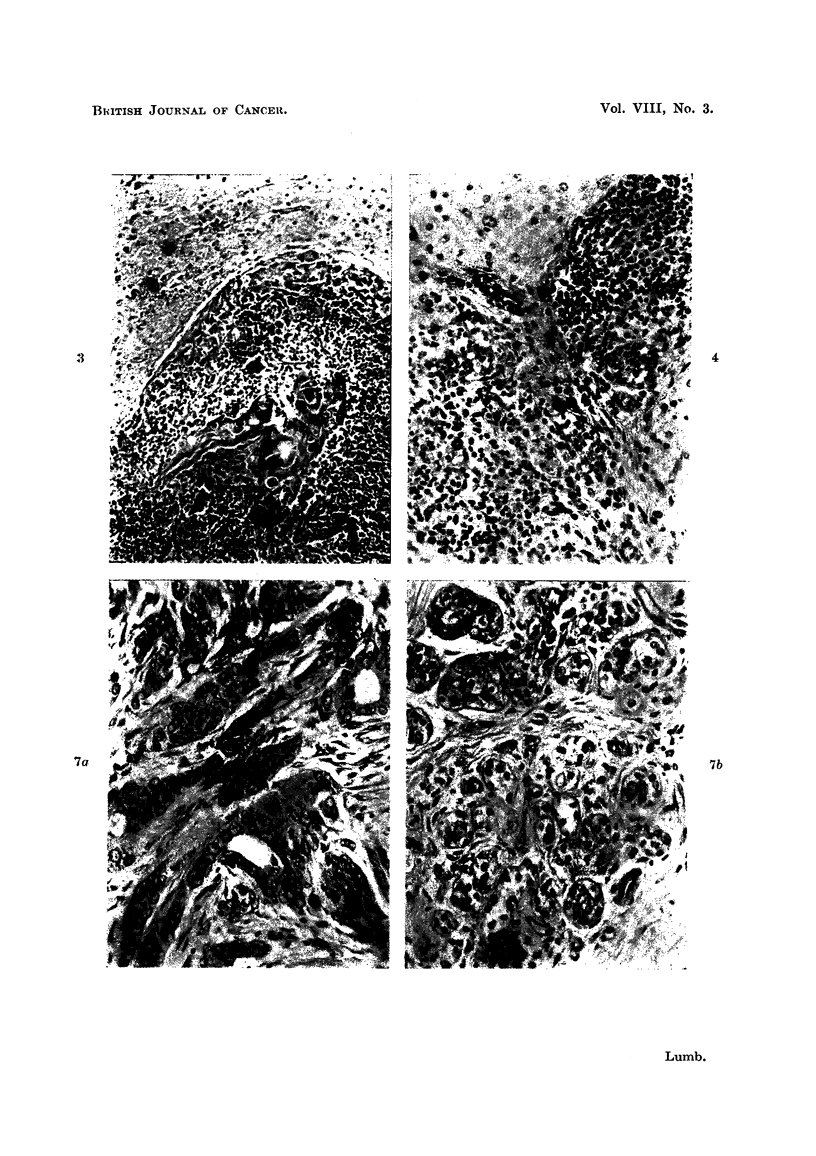

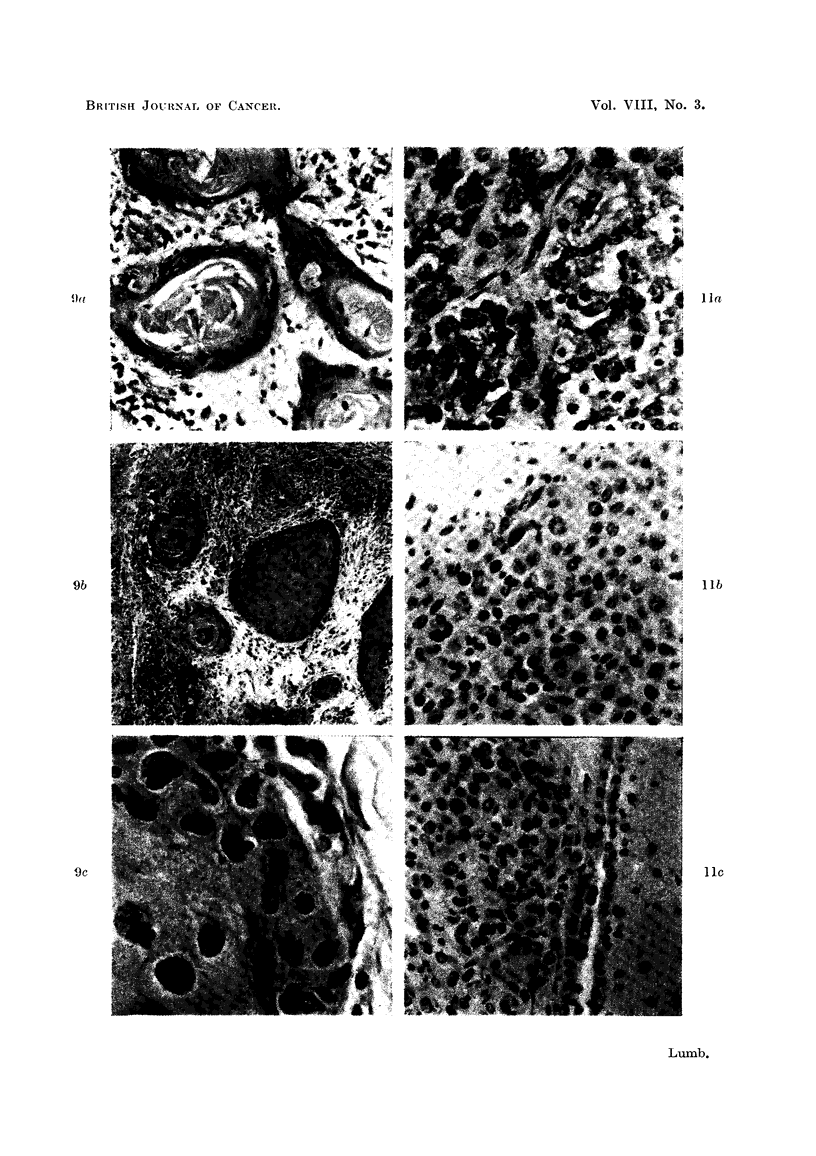

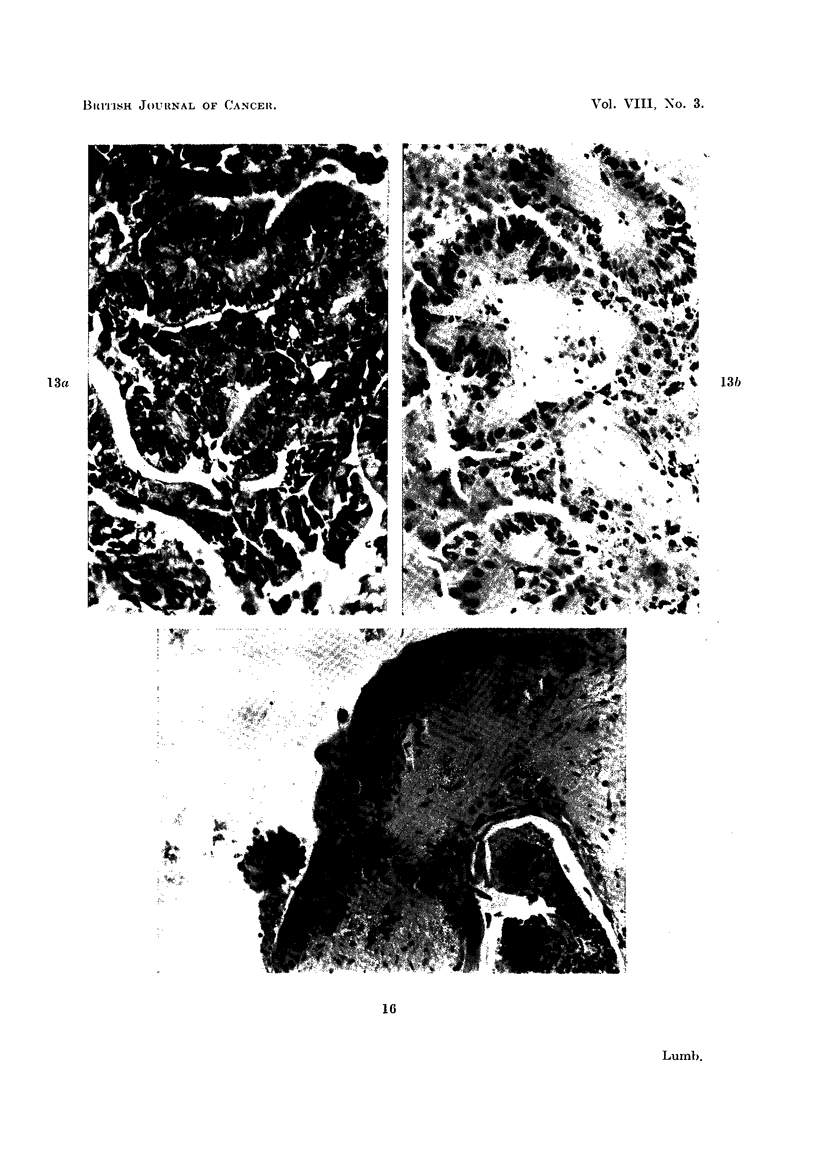

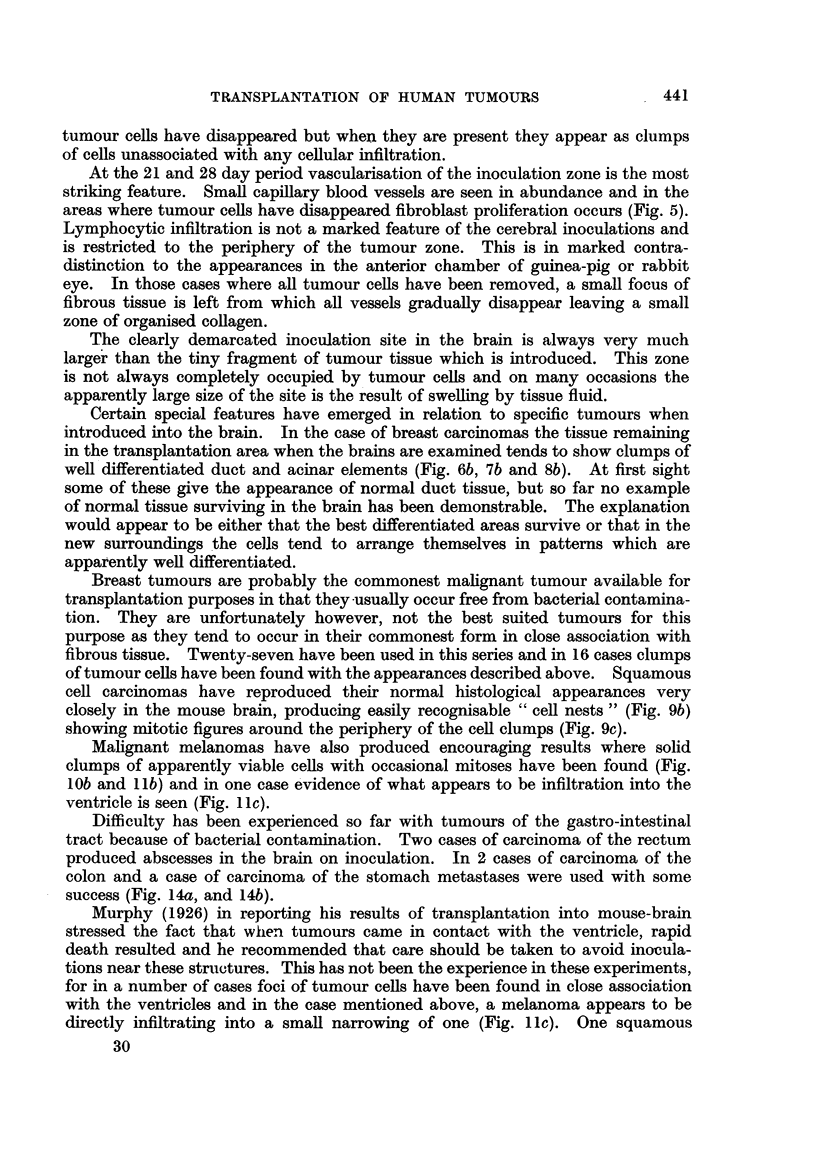

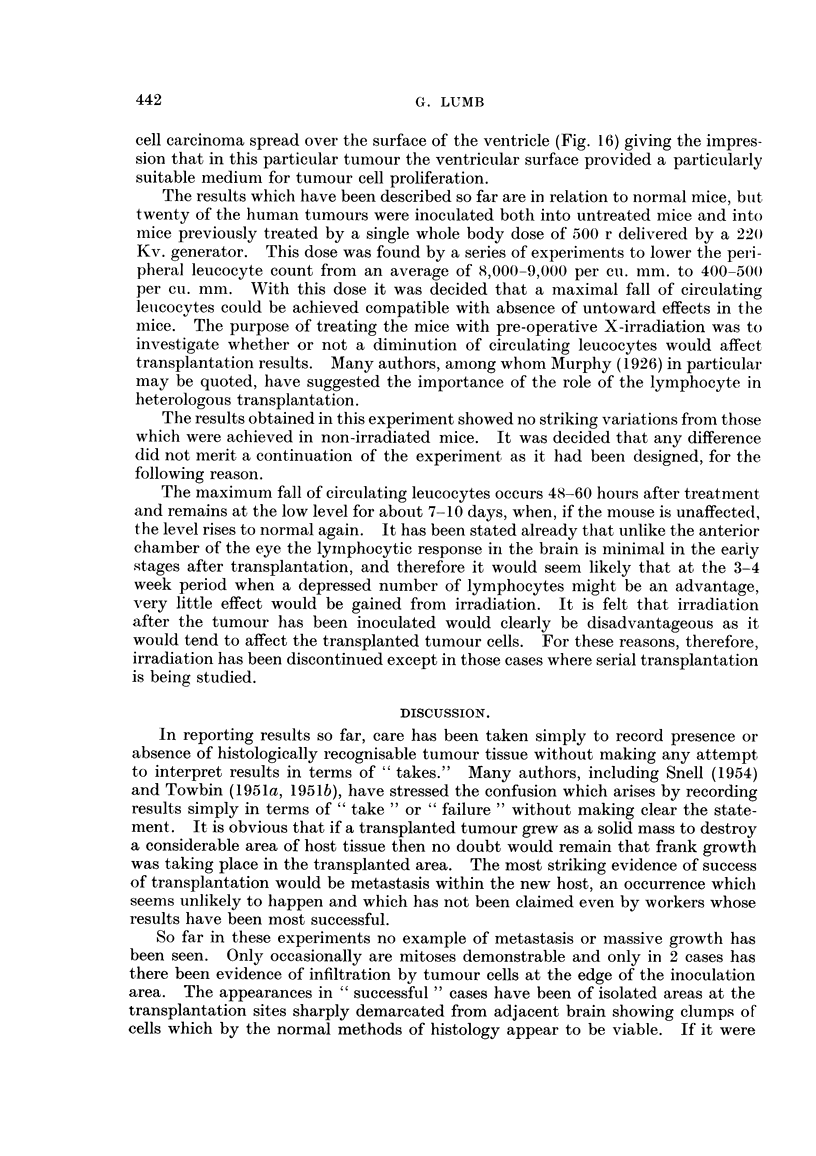

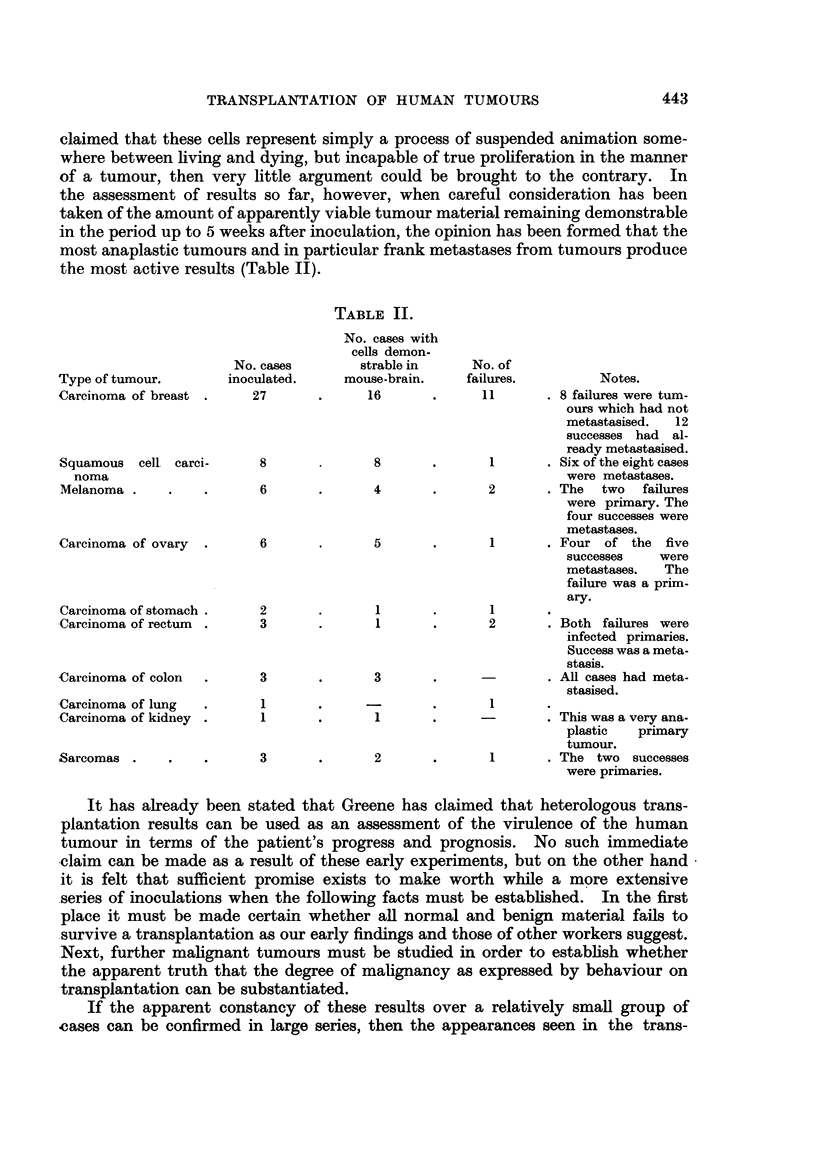

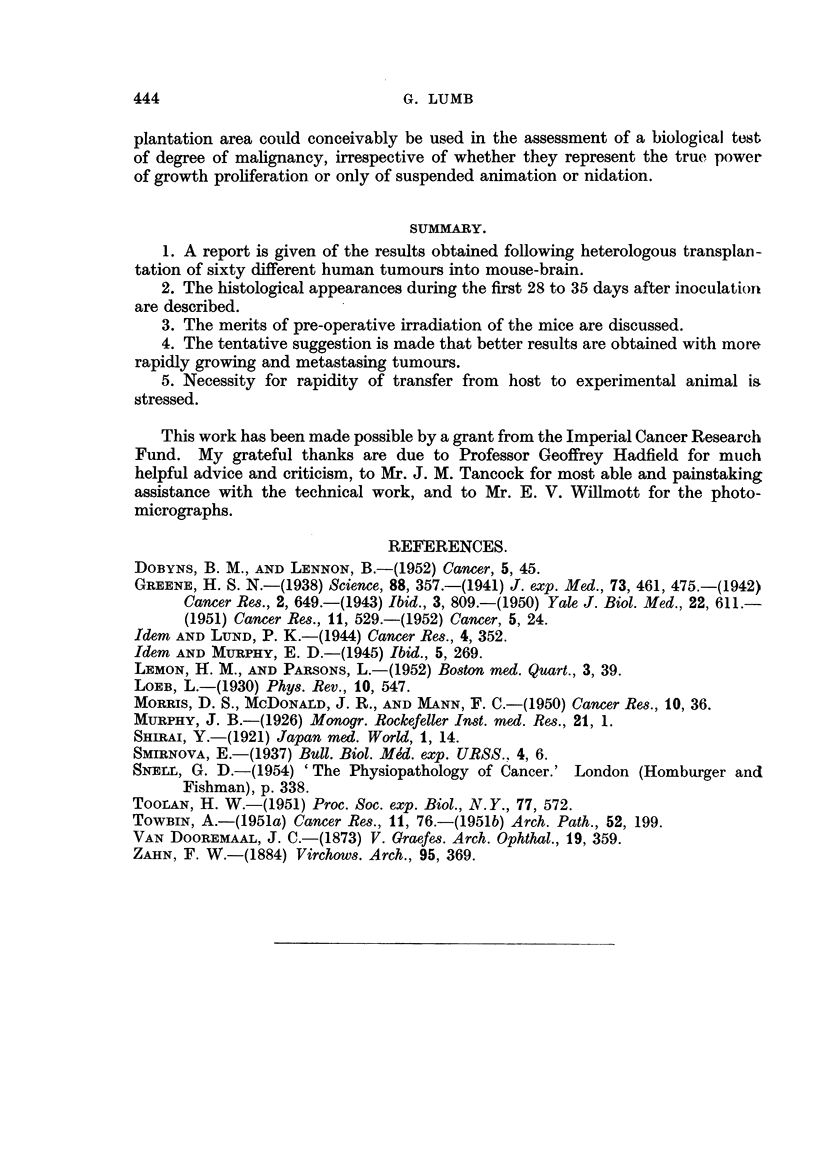


## References

[OCR_01322] GREENE H. S. N. (1950). The heterologous transplantation of human melanomas.. Yale J Biol Med.

